# Feasibility, Acceptability, and Appropriateness of an 8-Week Supervised Aerobic Exercise Intervention for Adults With Epilepsy: Pilot Randomized Controlled Trial

**DOI:** 10.2196/97392

**Published:** 2026-10-09

**Authors:** Sydney Churchill, Jeni Lansing, Angelique Brellenthin, Alison Phillips, Elizabeth Stegemöller, Jacob Meyer

**Affiliations:** 1 Department of Exercise and Sport Science University of North Carolina at Chapel Hill Chapel Hill, NC United States; 2 Department of Kinesiology University of Wisconsin–Madison Madison, WI United States; 3 Department of Kinesiology Iowa State University Ames, IA United States; 4 Department of Psychology Iowa State University Ames, IA United States

**Keywords:** epilepsy, feasibility, acceptability, aerobic exercise, intervention

## Abstract

**Background:**

Epilepsy affects multiple dimensions of health. Exercise may serve as a nonpharmacological strategy to improve health outcomes among adults with epilepsy; however, translating this potential into effective and scalable interventions remains challenging. Before advancing to efficacy trials, early-stage research is needed to establish whether exercise interventions are feasible, acceptable, and appropriate for this population.

**Objective:**

This study aimed to evaluate the feasibility, acceptability, and appropriateness of an 8-week supervised aerobic exercise intervention for adults with epilepsy, guided by the Obesity-Related Behavioral Intervention Trials (ORBIT) model.

**Methods:**

Adults with epilepsy aged 18-65 years were recruited between April 2024 and December 2024 and randomized to either a supervised aerobic exercise intervention (n=4) or a control group (n=2). The exercise group completed an 8-week supervised aerobic exercise program consisting of 50-minute sessions performed 3 days per week. The intervention incorporated behavior change techniques, including goal setting, action planning, habit formation, and commitment contracts to support exercise adherence. Process feasibility outcomes included recruitment, retention, adherence, survey completion, protocol compliance, and safety. Self-reported feasibility, acceptability, and appropriateness were assessed using the Feasibility of Intervention Measure, Acceptability of Intervention Measure, and Intervention Appropriateness Measure.

**Results:**

Overall, 34 individuals expressed interest in the study, 25 completed the screening survey, 10 were preliminarily eligible, and 6 were enrolled in the study over 8 months. Recruitment was low, with a recruitment rate of 0.75 participants per month and enrollment achievement of 16.7% of the target sample (target n=36). Retention and adherence were 100%. All exercise participants completed 24/24 sessions, with 100% weekly survey completion. Protocol compliance was high. No seizures occurred during exercise sessions. Mean scores for self-reported feasibility, acceptability, and appropriateness were high (>4 out of 5).

**Conclusions:**

The results of this Phase IIb pilot study suggest that a supervised aerobic exercise intervention for adults with epilepsy is feasible, acceptable, and appropriate to implement among participants who enroll. Recruitment remains the primary challenge to progressing this line of research and may represent a broader field-level barrier. Future trials should focus on improving how adults with epilepsy are reached and engaged in exercise research while preserving intervention strategies that support strong engagement among those who enroll.

## Introduction

Epilepsy is a seizure disorder that affects an estimated 52 million people worldwide [1]. The primary treatment approach to epilepsy is biomedical, with antiepileptic drugs serving as the frontline option for seizure control [2]. However, epilepsy is increasingly recognized as a multifaceted disorder that impacts physical, psychological, social, and emotional health [3-6], necessitating a biopsychosocial approach to care [7,8]. Consequently, there is a growing interest in exploring nonpharmacological, complementary treatment strategies, such as exercise, to potentially influence seizure-related outcomes and simultaneously improve additional health outcomes associated with epilepsy.

Aerobic exercise training offers a range of benefits for physical and mental health, such as improved cardiovascular fitness, reduced symptoms of depression and anxiety, and better sleep quality [9]. These benefits are particularly valuable for adults with epilepsy, who often experience a combination of physical and mental comorbidities [4,10]. Additionally, aerobic exercise may help reduce seizures by improving common seizure triggers, such as stress and sleep [11-13]. A consensus statement from the International League Against Epilepsy (ILAE) suggests that exercise is generally safe and may be beneficial for adults with epilepsy [14]. Taken together, exercise has the potential to serve as a complementary nonpharmacological strategy for managing epilepsy. However, despite these potential benefits, adults with epilepsy remain significantly less active than the general population [15,16], leaving substantial room for increasing activity and realizing the benefits in this population.

While exercise has shown promise for improving health and clinical outcomes in adults with epilepsy [17], translating this potential into consistent and scalable interventions remains a challenge. Recent intervention research has begun to address the feasibility of delivering physical activity interventions to adults with epilepsy, including remotely delivered approaches [18]; however, the existing evidence base is still limited, and current studies differ widely in design, participant characteristics, and exercise protocols. A small number of interventions have reported preliminary benefits, including improvements in physical health [19-23], mood and quality of life [21], reduced seizure frequency or no seizures occurring during exercise [20-22,24], and better memory [25] and executive functioning [26]. However, many of these studies include methodological constraints that limit the ability to conclude the efficacy of exercise for epilepsy [27-29], such as enrolling participants who are primarily seizure-free, allowing changes in group assignment after randomization, relying on unsupervised and self-reported physical activity, and using varying exercise prescriptions and outcome measures. Progress in the field requires a more structured approach to evaluating exercise interventions in this population before advancing to efficacy trials.

The Obesity-Related Behavioral Intervention Trials (ORBIT) model provides a systematic framework for addressing behavioral intervention challenges by emphasizing the importance of early-stage research to evaluate intervention feasibility and acceptability before advancing to larger efficacy trials [30]. Applying this framework to exercise interventions for adults with epilepsy can help ensure that future efficacy trials are built upon interventions that are practical to deliver, acceptable to participants, and appropriate for this population. Within the ORBIT model, Phase IIb research focuses on pilot and feasibility testing to evaluate whether an intervention protocol can be successfully implemented and to inform the design of future efficacy trials. Therefore, the primary aim of this study was to conduct a Phase IIb trial to evaluate the feasibility, acceptability, and appropriateness of a supervised 8-week aerobic exercise intervention for adults with epilepsy. Physical and psychological health outcomes were also assessed descriptively at baseline and study completion.

## Methods

### Study Design

This study, titled the EpiFIT Study, was an 8-week, parallel-group, randomized controlled pilot feasibility trial that adhered to CONSORT (Consolidated Standards of Reporting Trials; Multimedia Appendix 1) [31] and the CONSORT pilot and feasibility (Multimedia Appendix 2) [32] guidelines. The overall study design consisted of a screening process, a baseline visit (week 1), an 8-week intervention, and a final visit (week 10). The pilot trial was completed as planned at the conclusion of the prespecified study period. In-person visits and exercise sessions were conducted at a private research facility at Iowa State University. As the primary outcomes were feasibility, acceptability, and appropriateness, the study did not meet the criteria for the ClinicalTrials.gov Applicable Clinical Trial (ACT) Checklist and was not registered.

### Recruitment and Participants

Participants were recruited between April 2024 and December 2024 using a multifaceted approach, including tabling at community and campus events, distributing flyers around the community and the university, direct engagement at local sporting events, and via social media platforms. Additionally, mass emails were sent to alumni, faculty, staff, and students at multiple time points. Recruitment was further supported through partnerships with local hospitals and community-based epilepsy organizations, which facilitated referrals from patients and community networks.

Inclusion criteria were: adults (aged 18-65 years) with doctor-diagnosed epilepsy who had at least 1 seizure in the past year or were taking medication to control seizures, could provide physician’s consent to participate, were on stable seizure medication for ≥28 days prior to enrollment, were not currently meeting the recommended aerobic guidelines from the Physical Activity Guidelines for Americans [9], and were not participating in a structured exercise program. Exclusion criteria were based on recommendations made by the National Institute of Neurological Disorders and Stroke [33]. Participants were excluded from the study if they were taking >3 concomitant antiepileptic drugs, had status epilepticus within the past 2 years, had a neurostimulation device implanted or activated <1 year prior to enrollment (or a battery life unit that would not extend the duration of the trial), had epilepsy surgery <1 year prior to enrollment, had seizures that were triggered by exercise, drank alcohol excessively (defined as >7 per week for women and >14 per week for men [34]), smoked cigarettes, used cannabis, and were pregnant.

### Procedures

#### Screening and Baseline Visit

Interested participants completed an online screening survey and a phone screening survey to preliminarily assess eligibility. Potentially eligible participants scheduled a baseline visit. Participants were asked to abstain from caffeine 4 hours prior to the baseline visit to minimize its potential effects on heart rate during the submaximal exercise test. The baseline visit began with participants providing written informed consent. Participants then completed several electronic questionnaires, including a demographic and general health history questionnaire assessing participant characteristics and self-reported comorbid conditions; an epilepsy history questionnaire; the Patient Health Questionnaire-9 (PHQ-9) [35]; the Generalized Anxiety Disorder-7 (GAD-7) [36]; the Pittsburgh Sleep Quality Index (PSQI) [37]; the Quality of Life in Epilepsy Inventory-31 (QOLIE-31) [38]; the Epilepsy Stigma Scale [39]; the Self-Reported Habit Index [40]; and the New General Self-Efficacy Scale [41]. Participants also completed a self-reported seizure diary at baseline and weekly throughout the intervention via REDCap (Vanderbilt University). Participants reported whether they experienced a seizure during the previous week, and if applicable, reported the number, timing, duration, type, and potential triggers of seizures using questions adapted from the Seizure Tracker logbook (Seizure Tracker LLC).

Following this, participants completed a seizure action plan from a template created by the Epilepsy Foundation of America with research staff to document their safety plan, emergency contacts, and seizure triggers. Next, height, weight, and body composition (via a handheld Bioelectrical Impedance Analyzer, Omron Healthcare Inc) were collected.

Finally, participants completed the Astrand-Rhyming Cycle Ergometer Test to estimate cardiorespiratory fitness (ie, VO_2_ max; [42]). The submaximal exercise test was conducted on a cycle ergometer (Lode B.V.) and lasted approximately 10 minutes. Participants wore heart rate monitors (Polar H10, Polar Electro Inc) and were asked to complete a 2-minute warm-up and to cycle at 50 revolutions per minute for 6 minutes at a workload aimed at obtaining a steady-state heart rate between 125 and 170 bpm. Heart rate was obtained at the fifth and sixth minute of the test and averaged to estimate the maximal aerobic capacity.

After the submaximal exercise test, participants were randomized to either the exercise group or control group using a 1:1 allocation ratio and a blocked randomization scheme (blocks of 4) based on a computer-generated allocation sequence created by JL and uploaded into REDCap. SC enrolled participants and initiated randomization through REDCap, which revealed group assignment based on the prespecified allocation sequence. Study personnel conducting intervention visits were aware of group assignments, as blinding was not feasible due to the nature of the intervention (ie, participants in the exercise group actively engaged in supervised sessions, whereas control participants did not). Participants were instructed to continue their usual medical care and prescribed epilepsy treatments throughout the study.

### Intervention

#### Exercise Group

The aerobic exercise training was delivered onsite, on a 1:1 basis. Several behavioral change techniques (BCTs) were used throughout the intervention to promote engagement and adherence. BCTs were coded using the BCT Taxonomy v1, a standardized, hierarchical classification of 93 discrete techniques used in behavior change interventions [43]. Participants came to the laboratory 3 days a week for 8 weeks for a total of 24 supervised exercise sessions. Each aerobic exercise session lasted 50 minutes and consisted of a 5-minute warm-up followed by a 40-minute exercise and a 5-minute cool-down. Participants had the choice to exercise on a treadmill, an elliptical, or a stationary bicycle, with instruction provided by the research team on how to use the machines (BCT taxonomy 4.1 – goals and planning: instruction on how to perform the behavior). The exercise prescription increased in intensity over time (BCT taxonomy 8.7 – repetition and substitution: graded tasks). Exercise intensity was prescribed using the Borg Rate of Perceived Exertion (RPE) scale, a validated tool that is commonly used to prescribe and monitor aerobic exercise intensity [44-46]. Exercise intensity RPE targets were based on the American College of Sports Medicine’s guidelines for exercise testing and prescription [47], with participants instructed to exercise at an RPE of 9-11 in week 1, RPE of 12-13 on weeks 2 and 3, and an RPE of 14-15 on weeks 4 through 8. Heart rate was collected to provide additional monitoring that participants were exercising within their prescribed intensity. They could either wear their own device (eg, using a smartwatch) or use one provided for them (Polar H10). Heart rate and RPE were collected every 2 minutes during the warm-up and cool-down and every 5 minutes during the exercise, and participants received feedback to increase or decrease their exercise intensity if heart rate fell outside of their target zone (BCT taxonomy 2.6 – feedback and monitoring: biofeedback). Participants could listen to music, watch television, or use their phones during the workout.

At the first exercise session, participants completed an online module created by the research team to enhance adherence to the intervention, focused on identifying and building intrinsic motivation and understanding habits and habit formation. Specifically, participants were asked to identify their motivations for starting aerobic exercise training (BCT taxonomy 1.1 –goals and planning: goal setting [behavior]) and to regularly discuss any intrinsic motivations with close friends and family members over the course of the intervention (BCT taxonomy 3.1 – social support: social support [unspecified]). Next, they were provided information regarding preparatory habit development (BCT taxonomy 8.3 – repetition and substitution: habit formation) and guided in creating preparatory action plans (BCT taxonomy 1.4 – goals and planning: action planning) and coping plans (BCT taxonomy 1.2 – goals and planning: problem solving) directly related to attending their aerobic exercise sessions [48]. Finally, they completed a commitment check (BCT taxonomy 1.9 – goals and planning: commitment) and contract (BCT taxonomy 1.8 – goals and planning: behavioral contract), in which they were asked to report their current level of commitment (on a 0-100 scale) to the 8-week intervention and to coming to their next exercise session, responded to questions related to what they needed to increase their level of commitment using the following question: “Will you attend your next aerobic exercise training session?” (yes or no response buttons), and then provided an electronic signature confirming they would [49].

At the first session of each subsequent week, participants electronically completed weekly reflections over motivation (free response), reassessed their habits and coping plans, and completed a commitment contract to attend their next session (identical to the baseline contract). Participants also completed a seizure diary before starting their exercise. Finally, the research team created an incentive board where participants moved pushpins after each exercise session (BCT taxonomy 10.1 – reward and threat: material incentive [behavior]). When participants reached a certain milestone, they were rewarded with an incentive prize consisting of a study hat, t-shirt, bag, and water bottle (BCT taxonomy 10.2 – reward and threat: material reward [behavior]).

#### Control Group

Participants in the control group were instructed to complete a seizure diary for 8 weeks, which was administered as a REDCap link via email at the beginning of the week. Three follow-up reminder emails were sent to participants throughout the week to complete the seizure diary.

#### Final Visit

At the final visit conducted after the 8-week intervention period, participants completed similar procedures to those at baseline. These included completing a seizure diary and the same questionnaires given at baseline with the addition of the self-reported Feasibility of Intervention Measure (FIM), Acceptability of Intervention Measure (AIM), and Intervention Appropriateness Measure (IAM) [50]. Final weight and body composition were collected. Participants ended the visit with the submaximal exercise test.

Participants in the control group were given the same online module to enhance exercise adherence that was provided to the exercise group and a binder of the intervention. The binder included safety information on exercising with epilepsy from the Epilepsy Foundation of America, information on what aerobic activity is and how to develop an exercise prescription, the Borg RPE Scale with instructions on how to use it, instructions on how to find manual heart rate and other ways to take heart rate, a blank calendar, and their exercise prescription personalized to their target heart rate reserve.

### Primary Outcome Measures: Process Feasibility and Self-Reported Implementation Outcomes

Process feasibility was assessed using study-level indicators of recruitment, retention, adherence, survey completion, protocol compliance, and safety. Recruitment was assessed through screening and recruitment rates. A single-item question on the screening survey asked participants how they heard about the study. The screening rate was calculated as the number of completed screening surveys divided by the total months of recruitment (8 months). Recruitment strategies are summarized as counts and percentages. The recruitment achievement rate was calculated by dividing the number of enrolled participants by the target sample size. Recruitment rate was calculated as the number of enrolled participants divided by the total months of recruitment.

Retention rate was defined as the percentage of enrolled participants who completed all study visits. Adherence was measured by recording attendance at study visits and exercise sessions. Adherence rate was defined as the percentage of completed study visits, including baseline and final assessments for all participants (exercise and control groups), as well as exercise sessions for the exercise group. Weekly survey completion was also monitored as a process feasibility indicator. All participants completed a weekly seizure diary, while exercise group participants additionally completed intervention-related check-ins assessing motivation, habit development, coping plans, and commitment to upcoming exercise sessions. Survey completion was calculated as the percentage of expected weekly surveys completed throughout the intervention.

Protocol compliance was assessed using 2 criteria, with priority given to ensuring safety by focusing primarily on participant completion of the full 50-minute exercise duration for each session. The secondary criterion was adhering to the prescribed RPE targets for each session, with participants encouraged to exercise at a pace they felt comfortable with if they could not reach the target RPE. Safety was monitored throughout the intervention period by recording adverse events, including seizure-related events (eg, seizure occurrence), as well as any participant-reported symptoms, complaints, or medical concerns that occurred during study participation. Seizure occurrence was also monitored using participants’ seizure diaries, which were reviewed weekly throughout the intervention period.

Self-reported intervention feasibility, acceptability, and appropriateness were assessed using 3 implementation outcome measures: the Feasibility of Intervention Measure (FIM, α=0.89), the Acceptability of Intervention Measure (AIM, α=0.85), and the Intervention Appropriateness Measure (IAM, α=0.91). These measures can be administered together or individually to evaluate participants’ perceptions of the intervention’s feasibility, acceptability, and appropriateness [50]. Each measure consists of 4 items rated on a 5-point Likert scale, where participants indicated their level of agreement with each statement (1=Completely Disagree to 5=Completely Agree). A total score for each domain was calculated by averaging the item responses, with a score of >3 indicative of intervention feasibility, acceptability, and appropriateness. Both groups completed all implementation outcome measures during their final visit.

### Exploratory Outcomes

#### Depression

Depressive symptoms were measured with the PHQ-9. The PHQ-9 is a 9-item measure scored on a 4-point Likert scale with 0 being “not at all” and 3 being “nearly every day.” Scores are categorized as 0-4, 5-9, 10-14, 15-19, and 20-27 as minimal, mild, moderate, moderately severe, and severe depression, respectively (α=0.89) [35].

#### Anxiety

The GAD-7 is used to assess clinical anxiety disorder in outpatient settings. The GAD-7 contains 7 items measured on a 4-point Likert scale with 0 being “not at all” and 3 being “nearly every day.” Scores are totaled together and can range from 0-21, with 0-4 indicating minimal anxiety, 5-9 indicating mild anxiety, 10-14 indicating moderate anxiety, and 15-21 indicating severe anxiety. The GAD-7 has strong internal consistency (α=0.92) [36].

#### Quality of Life

The QOLIE-31 is a multidimensional questionnaire that assesses quality of life in adults with epilepsy. This measure targets seven areas: emotional well-being, social functioning, energy and fatigue, cognitive functioning, seizure worry, medication effects, and overall quality of life. The internal consistency reliability for the measure ranged from 0.77 to 0.85. An overall score is obtained by weighting and summing the QOLIE-31 scale scores. The scores range from zero to 100, with a higher score indicating good quality of life [38].

#### Stigma

The Epilepsy Stigma Scale was adapted from the Parent Stigma Scale to measure stigma in adults with epilepsy. This is a 10-item assessment measured on a 7-point Likert scale ranging from 1 (Strongly Disagree) to 7 (Strongly Agree). It is scored by averaging items together, with higher scores reflecting higher perceived stigma. This measure has acceptable internal consistency (α=0.78) [39,51].

#### General Self-Efficacy

General self-efficacy was measured with the New General Self-Efficacy Scale. This scale is an 8-item measure with responses on a 5-point Likert scale ranging from 1 (Strongly Disagree) to 5 (Strongly Agree). Scores are calculated by averaging the items together, with higher scores reflecting higher self-efficacy (α=0.86-0.90) [41].

#### Sleep

Sleep quality was measured with the PSQI. This is a 19-item questionnaire measuring seven subcategories: subjective sleep quality, sleep latency, sleep duration, habitual sleep efficiency, sleep disturbances, use of sleeping medication, and daytime dysfunction. Each component score of the PSQI ranges from 0 to 3, with 3 indicating the greatest dysfunction or disturbance. The 7 component scores are then summed together to obtain a global PSQI ranging from 0 to 21, with higher scores indicating poorer sleep quality (α=0.83) [37].

#### Exercise Habits

Instigation and preparation habits were assessed using 4 items from the Self-Reported Behavioral Automaticity Index, which is a subset of items from the Self-Reported Habit Index [40,52,53]. The scale uses a 5-point Likert scale (eg, 1=Strongly disagree, 5=Strongly agree). Instigation habits were assessed with the statements: “Going for an aerobic exercise session is something I do automatically,” “Going for an aerobic exercise session is something I do without thinking,” and “Going for an aerobic exercise session is something I do without having to consciously remember.” Preparation habits were assessed with the statements: “Preparing what I need to do aerobic exercise is something I do automatically,” “Preparing what I need to do aerobic exercise is something I do without thinking,” and “Preparing what I need to go to do aerobic exercise is something I do without having to consciously remember.” Median scores for each subscale were computed, with higher scores indicating stronger habits.

### Sample Size Determination

Sample size was determined based on available resources, the study timeline, and published recommendations for pilot studies [30,54-56]. A target enrollment of 36 participants (18 per arm) was selected to estimate feasibility parameters.

### Statistical Analyses

All statistical analyses were conducted using R (version 4.1.1 or higher; R Core Team) [57]. Descriptive statistics summarized participant characteristics and study outcomes. Categorical variables were summarized using counts and percentages. Continuous variables were summarized using medians and ranges (minimum-maximum values). Self-reported feasibility, acceptability, and appropriateness measures (FIM, AIM, and IAM) were scored according to published procedures by averaging responses across the 4 items within each measure and were presented as mean (SD) scores to align with recommended scoring procedures. There was no missing data.

### Ethical Considerations

All study procedures were approved by Iowa State University’s Institutional Review Board (Approval number #23-375-00) and conducted in accordance with the Declaration of Helsinki. Written informed consent was obtained from all participants prior to enrollment. To ensure privacy and confidentiality, all study data were coded with unique identifiers, and identifying information was stored separately from research data on a password-protected computer. Participants did not receive compensation for their participation.

## Results

### Overview

Participant characteristics are presented in Table 1. The median duration of epilepsy diagnosis among participants was 10 years. All participants were taking antiepileptic drugs, with all 4 participants in the exercise group reporting polytherapy (ie, more than one antiepileptic drug) and both participants in the control group reporting monotherapy (ie, one antiepileptic drug). Five of 6 participants reported at least one seizure in the past year. Two participants had a typical seizure pattern of monthly seizures, 1 participant had a typical seizure pattern of yearly seizures, and 2 participants were unsure. Most participants had at least 2 comorbid conditions. None of the participants reported using a dietary approach to manage their seizures.

**Table 1:** Baseline demographic and clinical characteristics of participants with epilepsy.

Characteristics			Exercise (n=4)		Control (n=2)		Total (n=6)
Age (years), median (min-max)			26 (19-33)		28 (27-29)		28 (19-33)
Sex (Female), n (%)			1 (25)		2 (100)		3 (50)
Race (White), n (%)			4 (100)		2 (100)		6 (100)
Ethnicity (Hispanic), n (%)			0 (0)		1 (50)		1 (17)
Marital status (Married), n (%)			1 (25)		1 (50)		2 (33)
Employment, n (%)							
	Student	1 (25)		0 (0)		1 (17)	
	Part time	2 (50)		1 (50)		3 (50)	
	Full time	1 (25)		1 (50)		2 (33)	
Education, n (%)							
	Some college	2 (50)		0 (0)		2 (33)	
	College graduate (AD, BA, BS)	2 (50)		1 (50)		3 (50)	
	Graduate degree (MS, MA, PhD, MD)	0 (0)		1 (50)		1 (17)	
Number of comorbidities, n (%)^a^							
	0	1 (25)		0 (0)		1 (17)	
	1	0 (0)		0 (0)		0 (0)	
	2	1 (25)		0 (0)		1 (17)	
	3+	2 (50)		2 (100)		4 (67)	
Epilepsy history							
	Years diagnosed, median (min-max)	12.5 (2-15)		5 (2-8)		9.5 (2-15)	
Epilepsy type, n (%)^b^							
	Focal	2 (50)		0 (0)		2 (33)	
	Generalized	1 (25)		1 (50)		2 (33)	
	Combination^c^	0 (0)		1 (50)		1 (17)	
	Unknown	1 (25)		0 (0)		1 (17)	
Cause of seizures, n (%)							
	Unknown	4 (100)		2 (100)		6 (100)	
Frequency of seizures, n (%)							
	Daily	0 (0)		0 (0)		0 (0)	
	Weekly	0 (0)		0 (0)		0 (0)	
	Monthly	2 (50)		1 (50)		3 (50)	
	Yearly	1 (25)		0 (0)		1 (17)	
	Unsure	1 (25)		1 (50)		2 (33)	
Number of antiepileptic medications, n (%)							
	Monotherapy	0 (0)		2 (100)		2 (33)	
	Polytherapy	4 (100)		0 (0)		4 (67)	
	Diet to control seizures (yes), n (%)	0 (0)		0 (0)		0 (0)	

^a^Self-reported medical and psychiatric comorbidities included bowel disease, chronic low back pain, hearing impairment, depression, anxiety, gastroesophageal reflux disease, Tourette’s Syndrome, attention-deficit/hyperactivity disorder, obsessive-compulsive disorder, eating disorder, hyperinsomnia, pituitary microadenoma, pituitary cyst.

^b^Epilepsy type was self-reported.

^c^Combination of focal and generalized.

### Process Feasibility and Self-Reported Implementation Outcomes

Figure 1 displays the CONSORT diagram illustrating recruitment efforts and participant flow. The recruitment strategies used yielded 25 completed screening surveys in 8 months, with a screening rate of approximately 3 interested potential participants per month. Most potential participants were recruited via mass emails (20/25, 80%). Other recruitment strategies included word of mouth (2/25, 8%), social media posts (1/25, 4%), flyers in the community (1/25, 4%), and physician referral (1/25, 4%). Of the 25 participants who completed the screening survey, 10 potential participants were screened as potentially eligible based on the screener, and of those, 6 were enrolled in the study, achieving 16.7% of the target sample size (36 participants). Reasons for not enrolling the other 4 potential participants included unable to commute (n=2), not interested in the study (n=1), and inability to be contacted (n=1). The recruitment rate was approximately 0.75 enrolled participants per month.

**Figure 1:**
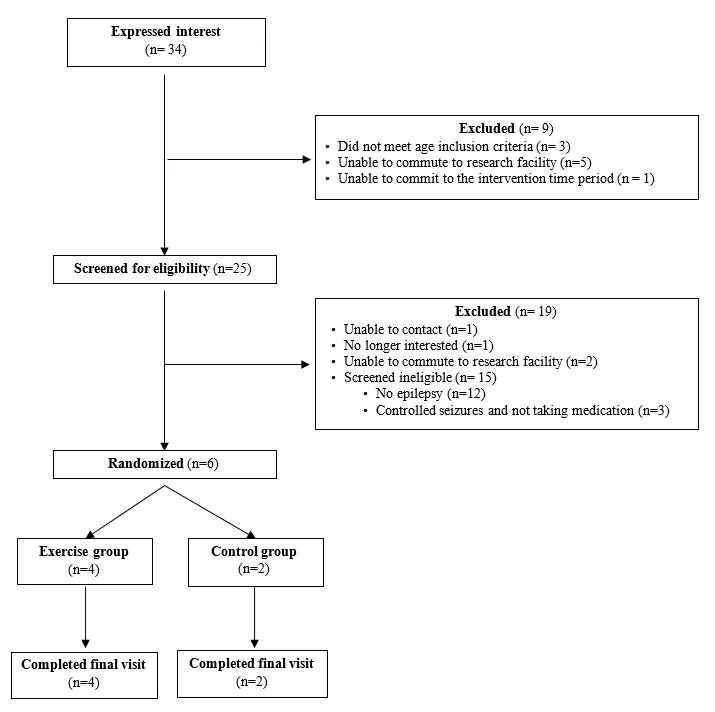
CONSORT (Consolidated Standards of Reporting Trials) diagram for EpiFIT intervention from recruitment to final visit.

All participants completed the assessment visits with a 100% retention rate. Out of the 108 study visits (1 baseline visit [both groups], 1 final visit [both groups], and 24 exercise sessions [exercise group]), all 108 were attended, for a study visit adherence rate of 100%. Weekly survey (ie, seizure diary [both groups] and health education check-ins [exercise group]) completion rate was 100% for both groups. Among exercise group participants, the median commitment score for attending their next scheduled exercise session was 100 (range 74-100). Protocol compliance for criterion 1 was met, as 100% of participants completed 24/24 sessions for the full 50-minute duration. Average weekly RPE met the target goal for each week: week 1 (mean 11, SD 0.81) was within the 9-11 range, weeks 2-3 (mean 12.2, SD 1.37 and mean 12.5, SD 0.79) were within the 12-13 range, and weeks 4-8 (mean 14, SD 0.85; mean 14.3, SD 0.42; mean 14.5, SD 0.57; mean 14.8, SD 0.5; and mean 14.8, SD 0.5) were within the 14-15 range. Total RPE compliance for all sessions was 88.54% (85/96 compliant). Deviations from the target RPE were minimal, with 6 sessions 1 unit and 5 sessions 2 units above or below the target goal. One adverse event was recorded during the intervention period. A participant fainted after skipping breakfast, which was attributed to low blood sugar. Following monitoring and physician approval, the participant was able to continue study participation.

No seizures occurred during the supervised exercise sessions. Of the 4 participants, one in the exercise group reported one seizure outside of the exercise sessions and both participants in the control group reported seizures, with a total of 5 seizures reported during the intervention period. When asked about the cause of the seizure, the participant in the exercise group reported missing medication. Other reasons for seizures in the control group included being overtired or having irregular sleep, emotional stress, alcohol or drug use, and fever or overheating.

Participants’ mean scores (out of a total score of 5) for the self-reported feasibility, acceptability, and appropriateness measures were 4.46 (SD 0.60), 4.33 (SD 0.66), and 4.42 (SD 0.80), respectively (Table 2).

**Table 2:** Self-reported feasibility, acceptability, and appropriateness of the EpiFIT intervention measured using the FIM, AIM, and IAM (n=6).

Measures			Mean (SD)^a^
Feasibility of Intervention Measures (FIM)			
	EpiFIT seems implementable	4.50 (0.55)	
	EpiFIT seems possible	4.67 (0.52)	
	EpiFIT seems doable	4.50 (0.55)	
	EpiFIT seems easy to use	4.17 (0.98)	
Acceptability of Intervention Measure (AIM)			
	EpiFIT meets my approval	4.33 (0.82)	
	EpiFIT is appealing to me	4.33 (0.52)	
	I like EpiFIT	4.50 (0.84)	
	I welcome EpiFIT	4.17 (0.75)	
Intervention Appropriateness Measure (IAM)			
	EpiFIT seems fitting	4.50 (0.84)	
	EpiFIT seems suitable	4.33 (0.82)	
	EpiFIT seems applicable	4.33 (0.82)	
	EpiFIT seems like a good match	4.50 (0.84)	

^a^All questions have a range of 1-5.

### Exploratory Outcomes

Table 3 presents descriptive changes in psychological and physical health outcomes from baseline to the final visit. In the exercise group, median depression and anxiety scores increased from baseline to final. Stigma, general self-efficacy, VO_2_ max, weight, BMI, and body fat percentage showed little change. Instigation habit scores increased. Preparation habit scores remained stable. Among QOLIE-31 domains, median seizure worry and cognitive function scores increased. Median medication effects scores decreased. The remaining domains showed little change. Individual participant-level data for all outcomes are provided in Multimedia Appendix 3.

**Table 3:** Exploratory baseline-to-final changes in psychological and physical health outcomes.

	Exercise (n=4)		Control (n=2)	
Variables	Baseline median (min-max)^a^	Final median (min-max)	Baseline median (min-max)	Final median (min-max)
Depression	2.5 (1-13)	7.5 (2-13)	15 (8-22)	9.5 (7-12)
Anxiety	3 (1-12)	6 (2-20)	15.5 (15-16)	12 (11-13)
Stigma	2.9 (1-4.7)	2.5 (1-5.6)	3.3 (3.2-3.4)	3.8 (3.6-3.9)
General self-efficacy	3.6 (2.5-4.1)	3.5 (3.4-4)	2.9 (1.8-4)	3.4 (3.1-3.8)
Sleep	5.5 (4-10)	7 (2-9)	9.5 (3-16)	10.5 (4-17)
VO_2_ max (ml/kg/min)	39.1 (21-54.4)	39.9 (23.9-50.2)	36.3 (32-40.7)	35.9 (32.8-39.1)
Weight (lbs)	175.5 (148.5-211.8)	172.9 (153.8-211.4)	136 (135-137)	130.2 (128-132.4)
BMI (kg/m^2^)	24.9 (22.1-36.8)	24.5 (22.9-37.4)	21.4 (21.4-21.5)	20.9 (20.1-21.6)
Body fat %	16 (10.2-41)	16.7 (11.3-42.1)	22.4 (21.9-22.9)	20.9 (18.9-22.9)
Habit Index				
Instigation subscale	1.5 (1-3)	3 (2-3.3)	1.3 (1-1.7)	1 (1-1)
Preparation subscale	3.5 (1-4)	3.3 (2.3-4.3)	1.5 (1.3-1.7)	1 (1-1)
QOLIE-31^b^				
Seizure worry	79.3 (68.7-95)	86.3 (28.7-96)	54.7 (36.7-72.6)	54.5 (27.3-81.7)
Overall QOL	72.5 (40-77.5)	71.3 (27.5-77.5)	57.5 (37.5-77.5)	63.8 (60-67.5)
Emotional well-being	58 (36-88)	58 (28-80)	40 (24-56)	56 (52-60)
Medication effects	54.2 (33.3-88.9)	23.6 (0-100)	66.7 (61.1-72.3)	69.5 (50-88.9)
Energy and fatigue	47.5 (30-75)	45 (10-55)	30 (10-50)	45 (35-55)
Cognitive function	36.2 (10-80)	43.6 (3.3-93.3)	24.3 (19.7-28.9)	37.6 (25.6-49.7)
Social function	93 (81-100)	92.5 (28-100)	65 (35-95)	78.5 (62-95)
Total QOLIE-31	64 (39.8-81.5)	63.7 (18.6-81.8)	44.2 (30.7-57.8)	55.6 (48.9-62.2)

^a^Data are presented as median (minimum-maximum) because of the small group sample size.

^b^QOLIE-31: Quality of Life in Epilepsy Inventory-31.

## Discussion

### Principal Findings

The primary aim of this Phase IIb study was to evaluate whether an 8-week supervised aerobic exercise intervention was feasible, acceptable, and appropriate for adults with epilepsy. Recruitment feasibility was poor; however, intervention implementation among enrolled participants was successful. All enrolled participants completed study visits and exercise sessions with no dropout, and survey completion and protocol compliance were high. No exercise-related seizures occurred during the supervised sessions. Participants also self-reported high perceptions of intervention feasibility, acceptability, and appropriateness. Overall, while recruitment strategies will require further refinement before progressing to a larger trial, the intervention protocol was well received by participants, supporting its continued development and evaluation in future studies.

### Comparison With Prior Work

Recruitment challenges are consistent with prior exercise interventions in adults with epilepsy, where enrollment has similarly fallen short of target samples [16] or exercise interventions have shown limited appeal in this population [58]. Possible contributors to recruitment challenges in this study include the lack of an on-site medical center at the university, which limited direct access to potential participants. Although efforts were made to collaborate with nearby hospitals, ongoing reminders were needed to sustain recruitment momentum. Additionally, the decision to make the intervention supervised was driven by a commitment to ensuring participant safety and proper execution of exercises. However, this decision excluded several potential participants who were interested in participating but were unable to commute, a commonly reported barrier to exercise intervention participation in this population [59]. Yet, a recent theory-based, remotely delivered, 12-week physical activity intervention for adults with epilepsy recruited through a Level 4 Epilepsy Center also fell short of its enrollment target (26/30) [18]. Together, these findings suggest that recruitment challenges in exercise research among adults with epilepsy may represent a broader field-level barrier rather than a problem specific to this study or intervention format. Addressing this barrier will likely require a more comprehensive approach to recruitment that combines stronger clinical partnerships, potentially across multiple sites, with accessible intervention delivery and a better understanding of how to engage adults with epilepsy in exercise research.

Although recruitment was poor, intervention implementation among participants was successful. Similar patterns have been reported across exercise interventions for adults with epilepsy, where recruitment rates were low (9% to 60%), but completion and adherence rates were high, ranging from 75% to 100% [29]. This pattern was also observed in the recent remote trial, which reported high retention, adherence, and acceptability despite falling short of its enrollment target [18]. In this study, participants demonstrated strong engagement throughout the intervention, with high retention, adherence, survey completion, and protocol compliance. Participants also self-reported that the intervention was feasible, acceptable, and appropriate, providing further support for the intervention among those enrolled. The use of BCTs, including goal setting, action planning, commitment contracts, feedback and monitoring, and habit formation strategies, may have supported participant engagement and adherence. Future efforts to move the field forward should focus on improving recruitment while preserving the intervention strategies that support engagement among those who enroll.

The exploratory outcomes should be interpreted cautiously given the small sample size, differences from findings reported in prior exercise intervention studies in adults with epilepsy [20,21,25,26], and contextual factors that may have influenced participant responses. For example, 3 of the 4 participants in the exercise group experienced challenging personal circumstances unrelated to the study during the week of their final assessment, which influenced their responses to the psychological and sleep measures. Additionally, baseline levels of depression and anxiety were higher in the control group than in the exercise group, providing greater opportunity for improvement among control participants. These factors, combined with the small sample size, make it difficult to draw meaningful conclusions about changes in exploratory outcomes.

### Limitations and Strengths

This study had several limitations. The most significant was the small sample size and difficulties with recruiting participants. Recruitment was limited to a convenience sample of individuals in the local rural area, who were primarily college-aged and non-Hispanic White. Additionally, epilepsy diagnosis was based on physician diagnosis rather than confirmation by an epileptologist or neurologist. Therefore, it is possible that individuals with seizure-like activity or other conditions that mimic epilepsy may have been included. Additionally, epilepsy type was also determined through self-report, which may have resulted in misclassification of epilepsy characteristics. Future studies should incorporate clinical verification of epilepsy diagnosis and subtype when feasible.

The study design also included several methodological limitations. Due to the nature of the intervention, participant and study personnel blinding was not feasible, which may have influenced participant responses and outcome reporting. Additionally, randomization resulted in baseline differences between groups, particularly in the psychological outcomes, which limited meaningful comparison of the exploratory outcomes. Further, exercise intensity was primarily prescribed and monitored using the Borg RPE scale. Although RPE is an established and commonly used proxy indicator for prescribing aerobic exercise intensity [45], it is a subjective measure and may be influenced by individual perception or prior exercise experience [60]. Additionally, participants were permitted to use either personal wearable devices or a Polar H10 heart rate monitor to monitor heart rate during exercise sessions. Because these devices use different methods to estimate heart rate, measurement accuracy may have varied across participants.

Despite these limitations, the study also had several strengths that can inform future exercise interventions in adults with epilepsy. The intervention incorporated a standardized supervised exercise protocol alongside multiple evidence-based behavior change techniques designed to support adherence. Feasibility was evaluated across multiple domains, including process feasibility study-level indicators (ie, recruitment, retention, adherence, weekly survey completion, protocol compliance, and safety) and self-reported feasibility, acceptability, and appropriateness measures. Collectively, these findings provide practical guidance for refining future exercise interventions in adults with epilepsy.

### Conclusions

The results of this Phase IIb pilot study suggest that a supervised aerobic exercise intervention for adults with epilepsy is feasible, acceptable, and appropriate to implement among participants who enroll. Using supervised exercise sessions, weekly preparatory habits and attendance-commitment checks, and flexibility within scheduling may be useful strategies to promote adherence among adults with epilepsy. Recruitment barriers remain the primary challenge to progressing this line of research and may represent a broader field-level barrier. Future trials should therefore focus on improving how adults with epilepsy are reached and engaged in exercise research, while preserving the intervention strategies that support strong engagement among those who enroll.

## Appendix

Multimedia Appendix 1 CONSORT checklist.

Multimedia Appendix 2 CONSORT pilot and feasibility.

Multimedia Appendix 3 Individual participant values for exploratory psychological and physical health outcomes measured at baseline (pre) and the final assessment (post).

## Abbreviations

ACT: Applicable Clinical Trial
AIM: Acceptability of Intervention Measure
BCT: Behavioral Change Technique
CONSORT: Consolidated Standards of Reporting Trials
FIM: Feasibility of Intervention Measure
GAD-7: Generalized Anxiety Disorder-7
IAM: Intervention Appropriateness Measure
ILAE: International League Against Epilepsy
ORBIT: Obesity-Related Behavioral Intervention Trial
PHQ-9: Patient Health Questionnaire-9
PSQI: Pittsburgh Sleep Quality Index
QOLIE-31: Quality of Life in Epilepsy Inventory-31
RPE: Rate of Perceived Exertion

## Data Availability

The data that support the findings of this study are available on request from the corresponding author.

## References

[1] Epilepsy Collaborators GBD (2025). Global, regional, and national burden of epilepsy, 1990-2021: a systematic analysis for the Global Burden of Disease Study 2021. Lancet Public Health.

[2] Kanner AM, Bicchi MM (2022). Antiseizure medications for adults with epilepsy: a review. JAMA.

[3] Kwon C, Jacoby A, Ali A, Austin J, Birbeck GL, Braga P, Cross JH, de Boer H, Dua T, Fernandes PT, Fiest KM, Goldstein J, Haut S, Lorenzetti D, Mifsud J, Moshe S, Parko KL, Tripathi M, Wiebe S, Jette N (2022). Systematic review of frequency of felt and enacted stigma in epilepsy and determining factors and attitudes toward persons living with epilepsy-Report from the International League Against Epilepsy Task Force on Stigma in Epilepsy. Epilepsia.

[4] Keezer MR, Sisodiya SM, Sander JW (2016). Comorbidities of epilepsy: current concepts and future perspectives. Lancet Neurol.

[5] De Giorgi R, Lomax T, Moreno C, Van Assche E, Osimo EF, Bajorek T, Sen A, Fazel S, Baune BT, Kandasamy R, Yu R, Peña-Ceballos J, Lattanzi S, Camazón PA, Del Rincon CR, Yogarajah M, Assenza G, Robinson T, Aledo-Serrano A, Cipriani A (2025). Epilepsy and mental health disorders: current challenges and potential solutions. Br J Psychiatry.

[6] Thijs RD, Surges R, O'Brien TJ, Sander JW (2019). Epilepsy in adults. Lancet.

[7] Elliott JO, Richardson VE (2014). The biopsychosocial model and quality of life in persons with active epilepsy. Epilepsy Behav.

[8] Mula M, Sander JW (2016). Psychosocial aspects of epilepsy: a wider approach. BJPsych Open.

[9] (2018). Physical Activity Guidelines for Americans, 2nd edition.

[10] Doherty AJ, Harrison J, Christian DL, Boland P, Harris C, Hill JE, Stephani A, Reed J, Duffield S, Marson TG, Clegg AJ (2022). The prevalence of comorbidities in epilepsy: a systematic review. British Journal of Neuroscience Nursing.

[11] Balamurugan E, Aggarwal M, Lamba A, Dang N, Tripathi M (2013). Perceived trigger factors of seizures in persons with epilepsy. Seizure.

[12] Ge A, Gutierrez EG, Wook Lee S, Shah S, Carmenate Y, Collard M, Crone NE, Krauss GL (2022). Seizure triggers identified postictally using a smart watch reporting system. Epilepsy Behav.

[13] Sherzai S, Cole C, Duke A, Ayub N, Sherzai D, Casassa C (2026). Lifestyle factors impacting epilepsy and seizures: a narrative review. Am J Lifestyle Med.

[14] Capovilla G, Kaufman K, Perucca E, Moshé SL, Arida R (2016). Epilepsy, seizures, physical exercise, and sports: a report from the ILAE task force on sports and epilepsy. Epilepsia.

[15] Vancampfort D, Ward PB, Stubbs B (2019). Physical activity and sedentary levels among people living with epilepsy: a systematic review and meta-analysis. Epilepsy Behav.

[16] Zhang J, Yu Y, Chen Z, Wang Y (2024). Trends and disparities in the prevalence of physical activity among US adults with epilepsy, 2010-2022. Epilepsy Behav.

[17] Arida RM (2023). Physical exercise as a strategy to reduce seizure susceptibility. Pharmacoresistance in Epilepsy From Genes and Molecules to Promising Therapies.

[18] Alexander HB, O'Connell N, Munger Clary HM, Burgos-Aguilar C, Peters C, Brubaker PH, Fountain NB, Shaltout HA, Fanning J (2026). A randomized feasibility pilot trial of a remotely delivered physical activity intervention for adults with epilepsy. Epilepsy Behav.

[19] Kumar M, Ramanujam B, Barki S, Dwivedi R, Vibha D, Singh RK, Tripathi M (2022). Impact of exercise as a complementary management strategy in people with epilepsy: a randomized controlled trial. Epilepsy Behav.

[20] Häfele CA, Rombaldi AJ, Feter N, Häfele V, Gervini BL, Domingues MR, da Silva MC (2021). Effects of an exercise program on health of people with epilepsy: a randomized clinical trial. Epilepsy Behav.

[21] McAuley JW, Long L, Heise J, Kirby T, Buckworth J, Pitt C, Lehman KJ, Moore J, Reeves AL (2001). A prospective evaluation of the effects of a 12-week outpatient exercise program on clinical and behavioral outcomes in patients with epilepsy. Epilepsy Behav.

[22] Åkerlund S, Varkey E, Klecki J, Zelano J, Ben-Menachem E (2021). Randomized controlled trial of moderate cardiovascular exercise for patients with drug-resistant epilepsy. Epilepsy Behav.

[23] Lakshmi ANR, Jayalakshmi S, SudhindraVooturi (2020). Evaluation of factors influencing obesity and the effect of a 12-week home-based exercise program in people with epilepsy - randomized control trial. Epilepsy Behav.

[24] Lundgren T, Dahl J, Yardi N, Melin L (2008). Acceptance and commitment therapy and yoga for drug-refractory epilepsy: a randomized controlled trial. Epilepsy Behav.

[25] Allendorfer JB, Brokamp GA, Nenert R, Szaflarski JP, Morgan CJ, Tuggle SC, Ver Hoef L, Martin RC, Szaflarski BA, Kaur M, Lahti AC, Bamman MM (2019). A pilot study of combined endurance and resistance exercise rehabilitation for verbal memory and functional connectivity improvement in epilepsy. Epilepsy Behav.

[26] Feter N, Alt R, Häfele CA, da Silva MC, Rombaldi AJ (2020). Effect of combined physical training on cognitive function in people with epilepsy: results from a randomized controlled trial. Epilepsia.

[27] Johnson EC, Helen Cross J, Reilly C (2020). Physical activity in people with epilepsy: a systematic review. Epilepsia.

[28] Sauls RM, Buro AW, Kirby RS (2024). Lifestyle behavioral interventions and health-related outcomes among people with epilepsy: a review of randomized controlled trials. Am J Health Promot.

[29] Duñabeitia I, Bidaurrazaga-Letona I, Diz JC, Colon-Leira S, García-Fresneda A, Ayán C (2022). Effects of physical exercise in people with epilepsy: a systematic review and meta-analysis. Epilepsy Behav.

[30] Czajkowski SM, Powell LH, Adler N, Naar-King S, Reynolds KD, Hunter CM, Laraia B, Olster DH, Perna FM, Peterson JC, Epel E, Boyington JE, Charlson ME (2015). From ideas to efficacy: the ORBIT model for developing behavioral treatments for chronic diseases. Health Psychol.

[31] Hopewell S, Chan A, Collins GS, Hróbjartsson A, Moher D, Schulz KF, Tunn R, Aggarwal R, Berkwits M, Berlin JA, Bhandari N, Butcher NJ, Campbell MK, Chidebe RCW, Elbourne D, Farmer A, Fergusson DA, Golub RM, Goodman SN, Hoffmann TC, Ioannidis JPA, Kahan BC, Knowles RL, Lamb SE, Lewis S, Loder E, Offringa M, Ravaud P, Richards DP, Rockhold FW, Schriger DL, Siegfried NL, Staniszewska S, Taylor RS, Thabane L, Torgerson D, Vohra S, White IR, Boutron I (2025). CONSORT 2025 statement: updated guideline for reporting randomised trials. Lancet.

[32] Eldridge SM, Chan CL, Campbell MJ, Bond CM, Hopewell S, Thabane L, Lancaster GA, PAFS consensus group (2016). CONSORT 2010 statement: extension to randomised pilot and feasibility trials. BMJ.

[33] Fertig E, Fureman BE, Bergey GK, Brodie MA, Hesdorffer DC, Hirtz D, Kossoff EH, LaFrance WC, Versavel M, French J, National Institute of Neurological DisordersStroke (2014). Inclusion and exclusion criteria for epilepsy clinical trials-recommendations from the April 30, 2011 NINDS workshop. Epilepsy Res.

[34] Patel AK, Balasanova AA (2021). Unhealthy alcohol use. JAMA.

[35] Kroenke K, Spitzer RL, Williams JB (2001). The PHQ-9: validity of a brief depression severity measure. J Gen Intern Med.

[36] Spitzer RL, Kroenke K, Williams JBW, Löwe Bernd (2006). A brief measure for assessing generalized anxiety disorder: the GAD-7. Arch Intern Med.

[37] Buysse DJ, Reynolds CF, Monk TH, Berman SR, Kupfer DJ (1989). The Pittsburgh sleep quality index: a new instrument for psychiatric practice and research. Psychiatry Res.

[38] Cramer JA, Perrine K, Devinsky O, Bryant-Comstock L, Meador K, Hermann B (1998). Development and cross-cultural translations of a 31-item quality of life in epilepsy inventory. Epilepsia.

[39] Austin J, Dunn D, Huster G, Rose D (1998). Development of scales to measure psychosocial care needs of children with seizures and their parents. J Neurosci Nurs.

[40] Verplanken B, Orbell S (2006). Reflections on past behavior: a self‐report index of habit strength. J Applied Social Pyschol.

[41] Chen G, Gully SM, Eden D (2001). Validation of a new general self-efficacy scale. Organizational Research Methods.

[42] Astrand I (1960). Aerobic work capacity in men and women with special reference to age. Acta Physiol Scand Suppl.

[43] Michie S, Richardson M, Johnston M, Abraham C, Francis J, Hardeman W, Eccles MP, Cane J, Wood CE (2013). The behavior change technique taxonomy (v1) of 93 hierarchically clustered techniques: building an international consensus for the reporting of behavior change interventions. Ann Behav Med.

[44] Borg G (1998). Borg's Perceived Exertion and Pain Scales.

[45] Chen MJ, Fan X, Moe ST (2002). Criterion-related validity of the Borg ratings of perceived exertion scale in healthy individuals: a meta-analysis. J Sports Sci.

[46] Borg G (1970). Perceived exertion as an indicator of somatic stress. Scand J Rehabil Med.

[47] American College of Sports Medicine (2026). ACSM's Guidelines for Exercise Testing and Prescription.

[48] Kaushal N, Rhodes RE, Spence JC, Meldrum JT (2017). Increasing physical activity through principles of habit formation in new gym members: a randomized controlled trial. Ann Behav Med.

[49] Halpern SD, Asch DA, Volpp KG (2012). Commitment contracts as a way to health. BMJ.

[50] Weiner BJ, Lewis CC, Stanick C, Powell BJ, Dorsey CN, Clary AS, Boynton MH, Halko H (2017). Psychometric assessment of three newly developed implementation outcome measures. Implement Sci.

[51] DiIorio C, Osborne Shafer P, Letz R, Henry T, Schomer DL, Yeager K, Project EASE Study Group (2003). The association of stigma with self-management and perceptions of health care among adults with epilepsy. Epilepsy Behav.

[52] Gardner B (2012). Habit as automaticity, not frequency. Eur Health Psychol.

[53] Orbell S, Verplanken B (2015). The strength of habit. Health Psychol Rev.

[54] Lancaster GA, Dodd S, Williamson PR (2004). Design and analysis of pilot studies: recommendations for good practice. J Eval Clin Pract.

[55] Julious SA (2005). Sample size of 12 per group rule of thumb for a pilot study. Pharmaceutical Statistics.

[56] Billingham SAM, Whitehead AL, Julious SA (2013). An audit of sample sizes for pilot and feasibility trials being undertaken in the United Kingdom registered in the United Kingdom Clinical Research Network database. BMC Med Res Methodol.

[57] (2018). The R project for statistical computing. The R Project.

[58] Alexander HB, Munger Clary HM, Shaltout HA, Fountain NB, Duncan P, Brubaker P, Fanning J (2024). Developing optimized physical activity interventions for drug-resistant epilepsy: challenges and lessons learned from a remote exercise intervention pilot trial. Epilepsy Behav Rep.

[59] Mitchell BS, Puzzo C, Morgan CJ, Szaflarski JP, Popp JL, Ortiz-Braidot R, Moyana A, Allendorfer JB (2023). Do people with epilepsy want to participate in an exercise intervention randomized controlled trial? - Results of a brief survey and its preliminary application. Epilepsy Behav Rep.

[60] Petro JL, Ferrari G, Cardozo LA, Vargas-Molina S, Carbone L, Kreider RB, Bonilla DA (2025). Validity of rating of perceived exertion scales in relation to movement velocity and exercise intensity during resistance-exercise: a systematic review. Sports Health.

