# Supplementary material for: Feasibility, Acceptability, and Appropriateness of an 8-Week Supervised Aerobic Exercise Intervention for Adults With Epilepsy: Pilot Randomized Controlled Trial
Source: JMIR Form Res. 2026 Oct 9;10:e97392. doi: 10.2196/97392 (PMC13695000; doi:10.2196/97392)
Supplement: Supplementary file 3 [file formative_v10i1e97392_app3.docx]

| **Table S1.** Individual participant values for exploratory psychological and physical health outcomes measured at baseline (Pre) and the final assessment (Post). | | | | | | |
| --- | --- | --- | --- | --- | --- | --- |
| **Outcome** | **EX^a^01** | **EX02** | **EX03** | **EX04** | **CON^a^01** | **CON02** |
| Depression^b^ (Pre) | 4 | 13 | 1 | 1 | 22 | 8 |
| Depression (Post) | 7 | 13 | 8 | 2 | 12 | 7 |
| Anxiety^c^ (Pre) | 4 | 12 | 1 | 2 | 15 | 16 |
| Anxiety (Post) | 6 | 20 | 6 | 2 | 13 | 11 |
| Stigma^d^ (Pre) | 3.2 | 4.7 | 2.5 | 1.0 | 3.2 | 3.4 |
| Stigma (Post) | 2.5 | 5.6 | 2.4 | 1.0 | 3.9 | 3.6 |
| General Self-Efficacy^e^ (Pre) | 3.3 | 2.5 | 3.9 | 4.1 | 1.8 | 4.0 |
| General Self-Efficacy (Post) | 3.4 | 3.5 | 4.0 | 3.5 | 3.1 | 3.8 |
| Sleep^f^ (Pre) | 4 | 5 | 10 | 6 | 16 | 3 |
| Sleep (Post) | 2 | 9 | 9 | 5 | 17 | 4 |
| VO_2_ Max^g^ (ml/kg/min) (Pre) | 28.9 | 21 | 54.4 | 49.4 | 32 | 40.7 |
| VO_2_ Max (ml/kg/min) (Post) | 37.2 | 23.9 | 50.2 | 48.8 | 32.8 | 39.1 |
| Weight (lbs) (Pre) | 179 | 212 | 172 | 149 | 137 | 135 |
| Weight (lbs) (Post) | 181 | 211 | 164 | 154 | 128 | 132 |
| BMI (kg/m^2^) (Pre) | 25.7 | 36.8 | 24.0 | 22.1 | 21.4 | 21.5 |
| BMI (kg/m^2^) (Post) | 26.0 | 37.4 | 23.0 | 23.0 | 20.1 | 21.6 |
| Body Fat % (Pre) | 15.9 | 41.0 | 16 | 10.2 | 21.9 | 22.9 |
| Body Fat % (Post) | 15.8 | 42.1 | 17.6 | 11.3 | 18.9 | 22.9 |
| **Habit Index^h^** |  |  |  |  |  |  |
| Instigation Subscale (Pre) | 1 | 1 | 3 | 2 | 1 | 1.7 |
| Instigation Subscale (Post) | 2 | 3.3 | 3.0 | 3.0 | 1 | 1 |
| Preparation Subscale (Pre) | 4 | 1 | 4 | 3 | 1 | 1 |
| Preparation Subscale (Post) | 4.3 | 2.3 | 3 | 3.7 | 1 | 1 |
| **QOLIE-31^i^** |  |  |  |  |  |  |
| Seizure Worry (Pre) | 95.0 | 67.6 | 69.3 | 89.3 | 72.7 | 36.7 |
| Seizure Worry (Post) | 90.0 | 28.6 | 82.6 | 96.0 | 81.7 | 27.3 |
| Overall Quality of Life (Pre) | 77.5 | 40.0 | 72.5 | 72.5 | 37.5 | 77.5 |
| Overall Quality of Life (Post) | 77.5 | 27.5 | 65 | 77.5 | 67.5 | 60 |
| Emotional Wellbeing (Pre) | 88 | 36 | 44 | 72 | 24 | 56 |
| Emotional Wellbeing (Post) | 80 | 28 | 48 | 68 | 52 | 60 |
| Medication Effects (Pre) | 44.4 | 33.3 | 63.9 | 88.9 | 72.3 | 61.1 |
| Medication Effects (Post) | 0 | 8.33 | 38.9 | 100 | 50 | 88.9 |
| Energy/Fatigue (Pre) | 40 | 30 | 55 | 75 | 10 | 50 |
| Energy/Fatigue (Post) | 50 | 10 | 40 | 55 | 35 | 55 |
| Cognitive Function (Pre) | 52.4 | 10 | 20 | 80 | 19.7 | 28.9 |
| Cognitive Function (Post) | 60.6 | 3.3 | 26.7 | 93.3 | 25.6 | 49.7 |
| Social Function (Pre) | 100 | 81 | 90 | 96 | 35 | 95 |
| Social Function (Post) | 100 | 28 | 85 | 100 | 62 | 95 |
| Total QOLIE-31 Score (Pre) | 72.9 | 39.8 | 55.1 | 81.5 | 30.7 | 57.8 |
| Total QOLIE-31 Score (Post) | 73.4 | 18.6 | 53.9 | 81.8 | 48.9 | 62.2 |
| ^a^EX = Exercise Group, CON = Control Group  ^b^Measured with the Patient Health Questionnaire-9  ^c^Measured with the Generalized Anxiety Disorder-7  ^d^Measured with the Epilepsy Stigma Scale  ^e^Measured with the New General Self-Efficacy Scale  ^f^Measured with the Pittsburgh Sleep Quality Index  ^g^VO_2_ max = estimated maximal oxygen uptake  ^h^Measured with the Self-Reported Behavioral Automaticity Index  ^i^Measured with the Quality of Life in Epilepsy Inventory-31 (QOLIE-31) | | | | | | |
